# INDICATORS OF ADIPOSITY ASSOCIATED WITH LOW BODY ESTEEM IN ADOLESCENTS

**DOI:** 10.1590/1984-0462/2020/38/2018383

**Published:** 2020-03-16

**Authors:** Ana Lúcia Viégas Rêgo, Rosangela Alves Pereira, Aldair José de Oliveira, Claudia Souza Lopes

**Affiliations:** a Instituto de Nutrição Josué de Castro, Universidade Federal do Rio de Janeiro, Rio de Janeiro, RJ, Brazil.; b Universidade Federal Rural do Rio de Janeiro, Seropédica, RJ, Brazil.; c Instituto de Medicina Social, Universidade do Estado do Rio de Janeiro, Rio de Janeiro, RJ, Brazil.

**Keywords:** Adolescents, Self-perception, Body mass index, Waist circumference, Obesity, Linear regression, Adolescentes, Autoimagem, Índice de massa corporal, Circunferência da cintura, Obesidade, Regressão linear

## Abstract

**Objective:**

To investigate the association between weight status and anthropometric indicators of adiposity with body esteem.

**Methods:**

Cross-sectional study including 305 adolescents from a public school in Rio de Janeiro, Brazil. Data were collected by a self-administered questionnaire and anthropometric measurements. The Body-Esteem Scale for Adolescents and Adults was used to evaluate total body esteem and the “appearance”, “weight”, and “attribution” domains. Body mass index (weight/stature^2^) was applied to assess weight status and waist circumference, the central body adiposity. The association between indicators of adiposity and body esteem was assessed using Student’s t-test or Mann-Whitney’s test and linear regression models, stratified by sex and age group.

**Results:**

Overweight/obesity was observed in 46% of younger adolescents (10 to 13 year-old girls, 10 to 14 year-old boys), 38% of older boys (15 to 18 year old), and 16% of older girls (14 to 18 year old). For both boys and girls in the younger age group, body mass index and waist circumference (as continuous variables) were inversely associated with total body esteem and weight domain. Overweight/obesity was associated with the appearance body esteem domain only among younger male adolescents; no association was found between either the body mass index or waist circumference and the attribution domain.

**Conclusions:**

Indicators of adiposity were associated with low body esteem. These findings underscore the fact that considering adolescents’ feelings concerning their body and appearance is important to promote a healthy control of weight.

## RESUMO

**Objetivo:**

Investigar a associação entre a condição de peso e os indicadores antropométricos de adiposidade com a estima corporal.

**Métodos:**

Estudo transversal com 305 adolescentes de uma escola pública do Rio de Janeiro, Brasil. Os dados foram coletados utilizando um questionário autoaplicado e por meio de medidas antropométricas. A Escala de Estima Corporal para Adolescentes e Adultos foi utilizada para avaliar a estima corporal total e os domínios “aparência”, “peso” e “atribuição”. O índice de massa corporal (peso/estatura^2^) foi utilizado para avaliar a condição do peso e circunferência da cintura, a adiposidade central. A associação entre indicadores de adiposidade e estima corporal foi avaliada por meio dos testes *t* de Student ou Mann-Whitney e por modelos de regressão linear, estratificados por sexo e faixa etária.

**Resultados:**

Sobrepeso/obesidade foi observado em 46% dos adolescentes mais jovens (meninas de 10 a 13 anos, meninos de 10 a 14 anos), em 38% dos meninos de 15 a 18 anos e em 16% das meninas de 14 a 18 anos. Na faixa etária mais jovem, o índice de massa corporal e a circunferência da cintura (como variáveis contínuas) foram inversamente associados à estima corporal total e ao domínio peso, tanto em meninos quanto em meninas. O sobrepeso/obesidade associou-se ao domínio corporal ‘aparência’ apenas entre os meninos mais jovens. Nenhuma associação foi encontrada entre índice de massa corporal e a circunferência da cintura e o domínio atribuição.

**Conclusões:**

Os indicadores de adiposidade foram associados à baixa estima corporal. Esses achados ressaltam a importância de se levar em consideração a percepção dos adolescentes em relação ao seu corpo e aparência na promoção do controle de peso saudável.

## INTRODUCTION

During adolescence, countless biopsychosocial changes contribute to an increased vulnerability to social and nutritional risks.^1^ ^,^ ^2^ One of these transformations concerns body esteem, which can be understood as an individual’s perception or feelings with regard to his/her body and appearance. It is an essential component in the formation of personality and affectivity.^3^ ^,^ ^4^

Nowadays, adolescents have to simultaneously cope with the changes intrinsic to this period of life and with the paradox of the increasing overweight/obesity prevalence^2^ ^,^ ^5^ allied to the mounting social pressure to attain the perfect body model, which is “extremely thin” for women and “muscular” for men.^6^ ^,^ ^7^ Hence, adolescents’ concerns about weight, body shape, and appearance may influence their body esteem and health conditions.^8^ The concept of body esteem has been signaled as more appropriate to investigate the effects of body dissatisfaction on adolescent’s health conditions as compared to self-esteem, which evaluates general aspects of the individual experience.^3^ Body esteem in adolescents has received academic attention, both to characterize its association with sex, age, and weight condition^8^ and to clarify its mediating role in more complex relationships, such as between body dissatisfaction and depression.^9^

Mendelson et al.^4^ developed and evaluated a scale to measure body esteem based on a self-administered questionnaire (Body Esteem Scale for Adolescents and Adults - BESAA), including questions on general feelings about appearance (“appearance” domain), weight satisfaction (“weight” domain), and evaluations attributed to others about one’s body and appearance (“attribution” domain). The authors observed that overweight adolescents who were concerned with body weight tended to score unfavorably in the “weight” and “appearance” domains. Moreover, other studies that applied the same questionnaire observed consistent findings, especially emphasizing the relation between body esteem and health.^8^ ^,^ ^10^ ^,^ ^11^

Furthermore, sex and age have been considered factors that strongly influence body esteem, especially among adolescents. Mendelson et al.^4^ evaluated 571 male and 763 female adolescents and young adults (between 12 and 25 years old) from Canada and observed that girls scored less than boys for total body esteem. Also using the BESAA, Ivarsson et al.^12^ noticed similar results in a study with 405 Swedish adolescents, among whom boys scored 63.5, on average, and girls, 55.0 (p=0.0001). Mak et al.^8^ also applied the BESAA in a study with 905 adolescents from Hong Kong and observed that boys tended to have higher total body esteem scores than girls (42.3 *versus* 40.1; p=0.001). Moreover, the total score increased with age in both boys and girls. These authors concluded that girls seemed to be more vulnerable to unfavorable body weight assessments than boys. Such finding was particularly evident among older adolescents with excess weight. In this group, older girls with excess weight were more prone to deficits in body esteem compared to their male counterparts.

Overall, excess weight has a negative effect on adolescents’ mental health. The associations of excessive weight with self-esteem,^13^ depression,^14^ shame and guilt,^15^ and anxiety^16^ have been well studied. Mental disorders are considered a public health problem in Brazil and are responsible for a significant portion of the burden of diseases in the country.^17^ ^,^ ^18^ In Brazil, despite the continuous increase in the prevalence of overweight/obesity among adolescents,^5^ only a few studies have already examined the association between weight status and body esteem in this population.^19^ ^,^ ^20^ This type of approach has the advantage of identifying target groups for intervention, in order to minimize stress situations related to overweight and body image. Given this context, this study aimed to investigate the association between weight status and anthropometric indicators of adiposity and body esteem in Brazilian adolescents, considering sex and age group strata.

## METHOD

This cross-sectional study was carried out in a public school in Rio de Janeiro, Brazil. All students aged 10 to 18 years-old enrolled in the 4^th^ to 9^th^ grades were included in the study. Exclusion criteria were: presenting any limitation to the anthropometric measurement, being under drug therapy to control weight, or being pregnant or during lactation. From the 326 eligible participants, 14 refused to participate in the study, four missed school during the period of data collection, and three did not provide all the required data. Therefore, the present study comprised 305 students (94% of the eligible participants) aged 10‒18 (174 boys, 131 girls).

Data were collected in classrooms from May to June of 2009 by means of a self-administered questionnaire. Additionally, body mass, height, and waist circumference (WC) measurements were taken in a separate room by a trained team using the protocol recommended by Lohman et al.^21^

Body esteem (total and appearance, weight, and attribution domains) was the dependent variable, whereas BMI and WC were the independent variables, and adolescents’ sex and age group were the covariables. The participants were divided into two age groups according to Chiara et al*.*,^22^ taking into account the differences in the process of growth, development, and sexual maturation of boys and girls: younger (boys aged 10 to 14; girls aged 10 to 13) and older (boys aged 15 to 18; girls aged 14 to 18) age groups.

Body esteem was assessed using the BESAA,^4^ which was transculturally adapted and validated for Brazilian adolescents.^19^ This scale comprises 23 questions with scores ranging over five response levels (never=0; almost never=1; sometimes=2; almost always=3; always=4). The instrument spans three subareas of feelings related to physical appearance (appearance), scoring 0 to 40; body satisfaction (weight), 0 to 32; and “how others see you” (attribution), 0 to 20. Scoring is estimated by subarea and overall (total score corresponds from 0 to 92). The higher the score, the better the adolescent’s body esteem.

Weight status was evaluated using the body mass index (BMI - body mass index (kg)/stature^2^ (m)), based on the World Health Organization criteria,^23^ and applying the percentile distribution by sex and age. Underweight was defined as BMI < percentile 3 of the reference distribution, normal weight as BMI between percentile 3 (inclusive) and percentile 85 (exclusive), and overweight/obesity as BMI ≥ percentile 85. Adolescents were classified as having high central adiposity if the WC was ≥ percentile 80 of the study group distribution, according to the criteria proposed by Taylor et al*.* ^24^

Scores for total body esteem and their three dimensions were treated as continuous variables; BMI and WC were treated as continuous and categorical as well. All analyses were stratified by sex and age group.

The Kolmogorov-Smirnov test was applied to verify the normality of distributions of dependent variables (BESAA scores). Differences in the BESAA score means across the categories of sex, age, weight status, and WC status were assessed using Student’s t-test (for total and weight body esteem) or Mann-Whitney’s test (for appearance and attribution body esteem).

Simple linear regression models adjusted by sex and age were developed to explore the association between body esteem and each of the independent variables (BMI and WC). These results were expressed as regression coefficients (β) and adjusted coefficients of determination (R^2^). The β values provide information on how far each unit value of an explanatory variable corresponded to the change brought about in each unit of the body esteem score (total and by domain). The adjusted R² values reveal the percentage variability of each association.^25^ The relation between explanatory variables and body esteem was considered significant at p<0.05.

This study was conducted according to the guidelines laid down in the Declaration of Helsinki, and procedures involving human subjects were approved by the Research Ethics Committee of Universidade do Estado do Rio de Janeiro (protocol No. 043.3.2006). A free informed consent form was signed by the adolescents and their guardians.

## RESULTS

Of the 305 studied adolescents, 57% (n=174) were boys; in the younger age group, boys comprised 71% (n=124) of sample, and in the older age group, 53% (n=50). Overall, girls presented lower mean scores than boys in the BESAA questionnaire for appearance (22.7 *versus* 25.7; p<0.01) and weight (18.8 *versus* 20.6; p=0.04) domains, and total body esteem (53.1 *versus* 57.2; p=0.03). Additionally, these differences were consistent in age categories for the appearance domain (Table 1).

**Table 1:** Means and standard deviations of body esteem scores of adolescents (n=305) from a public school by sex and age group, Rio de Janeiro, Brazil.

Variables	n	%	Body esteem scores							
			Appearance (0 to 40 points)		Weight (0 to 32 points)		Attribution^b^ (0 to 20 points)		Total^b^ (0 to 92 points)	
			Mean	SD	Mean	SD	Mean	SD	Mean	SD
Sex										
Male	174	57	25.7	7.2	20.6	7.3	10.9	4.3	57.2	15.3
Female	131	43	22.7	7.4	18.8	7.7	11.7	3.8	53.1	16.2
p-value^a^			<0.01		0.04		0.06		0.03	
Younger age group*										
Male	124	71	26.1	7.4	20.9	7.4	10.9	4.3	57.9	15.8
Female	69	29	23.2	7.5	19.2	7.3	11.4	3.6	53.7	15.6
p-value^a^			0.01		0.12		0.24		0.09	
Older age group**										
Male	50	53	24.9	6.8	19.7	7.1	11.0	4.2	55.6	14.0
Female	62	47	22.2	7.2	18.3	8.2	12.0	4.0	52.5	17.0
p-value^a^			0.03		0.34		0.18		0.30	

*: males: 10 to 14 years old; females: 10 to 13 years old; **: males: 15 to 18 years old; females: 14 to 18 years old; ^a^: Student’s t-test: total and weight body esteem; Mann-Whitney’s test: appearance and attribution body esteem; ^b^: Missing data: attribution and total body esteem scores: two cases; SD: standard deviation.

A very low underweight frequency was observed in the studied group (n=3), and these adolescents were excluded from the analysis. The distribution of weight status categories did not differ according to sex in the younger age group. However, in the older age group, 38.8% of the male and 16.4% of the female adolescents were overweight or obese (p<0.01; chi-square test). Boys presented greater frequency of elevated central adiposity than girls in both age groups (younger group - 29.8 *versus* 13.0%; p<0.01; older group - 24.0 *versus* 8.1%; p=0.02; chi-square test), as seen in Tables 2 and 3.

**Table 2:** Means and standard deviations of body esteem scores according to the classification of weight status^a^ and waist circumference^b^ by age group. Male adolescents (n=174)^¥^ of a public school in Rio de Janeiro, Brazil.

Variables	n	%	Appearance (0 to 40 points)		Weight (0 to 32 points)		Attribution (0 to 20 points)		Total (0 to 92 points)	
			Mean	SD	Mean	SD	Mean	SD	Mean	SD
Younger age group (10 to 14 years old)										
Weight status										
Normal weight	66	53.7	28.5	7.0	24.6	5.5	12.0	4.4	65.1	13.2
Overweight/obesity	57	46.3	23.4	7.4	16.7	7.1	9.7	3.8	49.8	14.6
p-value^c^			<0.01		<0.01		<0.01		<0.01	
WC status										
Normal	87	70.2	27.1	7.3	23.3	6.1	11.4	4.4	61.8	14.7
Elevated	37	29.8	23.5	7.1	15.4	7.2	9.6	3.7	48.5	14.6
p-value^c^			0.01		<0.01		0.02		<0.01	
Older age group (15 to 18 years old)										
Weight status										
Normal weight	30	61.2	25.2	6.4	22.5	6.9	10.6	4.5	58.4	14.1
Overweight/obesity	19	38.8	24.2	7.6	15.7	5.2	11.7	3.7	51.6	13.6
p-value^c^			0.37		<0.01		0.27		0.10	
WC status										
Normal	38	76.0	25.4	6.5	21.6	6.7	10.9	4.3	57.8	13.1
Elevated	12	24.0	23.5	2.3	14.0	5.0	11.3	4.0	48.8	15.1
p-value^c^			0.36		0.001		0.69		0.05	

^a^: Classified using the body mass index for age and sex percentile based on WHO^23^; ^b^: classified according to Taylor et al.^24^; ^c^: Student’s t-test: total- and weight-body esteem; Mann-Whitney’s test: appearance- and attribution-body esteem; ^¥^: underweight adolescents were excluded: two cases; WC: waist circumference; SD: standard deviation.

**Table 3:** Means and standard deviations of body esteem scores according to the classification of weight status^a^ and waist circumference^b^ by age group. Female adolescents (n=131)^¥^ of a public school in Rio de Janeiro, Brazil.

Variables	n	%	Appearance (0 to 40 points)		Weight (0 to 32 points)		Attribution^c^ (0 to 20 points)		Total^c^ (0 to 92 points)	
			Mean	SD	Mean	SD	Mean	SD	Mean	SD
Younger age group (10 to 13 years old)										
Weight status										
Normal weight	37	53.6	23.7	7.7	22.6	6.2	11.2	3.5	57.2	15.3
Overweight/obesity	32	46.4	22.6	7.5	15.3	6.6	11.7	3.8	49.7	15.2
p-value^d^			0.45		<0.01		0.52		0.05	
WC status										
Normal	60	87.0	23.6	7.5	20.2	7.0	11.6	3.6	55.2	15.3
Elevated	09	13.0	20.6	7.5	13.0	6.4	10.6	3.4	44.1	14.3
p-value^d^			0.25		0.01		0.57		0.05	
Older age group (14 to 18 years old)										
Weight status by BMI										
Normal weight	51	83.6	23.1	6.8	19.7	7.5	12.3	4.0	55.1	15.6
Overweight/obesity	10	16.4	17.6	8.0	11.5	8.7	11.0	3.8	40.1	19.8
p-value^d^			0.03		<0.01		0.23		0.01	
WC status										
Normal	57	91.9	21.9	7.0	18.2	8.0	11.9	3.9	51.9	16.2
Elevated	05	8.1	25.4	9.2	19.8	12.0	13.8	5.2	59.0	26.0
p-value^d^			0,47		0.68		0.41		0.38	

^a^: classified using body mass index (BMI) for age and sex percentile based on WHO^23^; ^b^: classified according to Taylor et al.^24^;^c^: missing data: attribution and total body esteem scores: two cases; ^d^: Student’s t-test: total and weight body esteem; Mann-Whitney’s test: appearance and attribution body esteem; ^¥^: underweight adolescent was excluded: one case; WC: waist circumference; SD: standard deviation.

In the younger age group, overweight/obese male adolescents showed significantly lower mean scores than those with normal weight for total body esteem and for the three analyzed domains. In the older age group, overweight/obese male adolescents presented lower mean scores for the weight domain than those with normal weight (15.7 *versus* 22.5; p<0.01). Comparable results were observed for the categories of WC status for both age groups (Table 2).

For younger female adolescents, differences between overweight/obesity and normal weight were observed only for the weight domain (15.3 *versus* 22.6; p<0.01). The same was observed for the categories of WC status (13.0 *versus* 20.2; p=0.01). For girls in the older age group, those with overweight or obesity had lower mean scores than normal weight girls for total body esteem (40.1 *versus* 55.1; p=0.01) and appearance (17.6 *versus* 23.1; p=0.03) and weight domains (11.5 *versus* 19.7; p<0.01). No differences in body esteem scores were observed for older female adolescents according to WC categories (Table 3).

BMI and WC showed significant inverse associations with body esteem in the weight domain for both age groups among male adolescents, while this association was observed among female adolescents only in the younger age group. BMI (β=-0.213; p=0.02) and WC (β=-0.276; p<0.01) showed an inverse association with the appearance domain among younger male adolescents and with total body esteem in the younger age groups for both boys (BMI - β=-0.310; p<0.01; WC - β=-0.383; p<0.01) and girls (BMI - β=-0.298; p=0.01; WC - β=-0.335; p=0.01). No association was seen between indicators of adiposity and body esteem scores for older girls (Table 4). In addition, indicators of adiposity explained variations in the weight body esteem scores for younger (BMI - R^2^=0.14; WC - R^2^=0.20) and older (BMI - R^2^=0.14; WC - R^2^=0.19) boys and younger girls (BMI - R^2^=0.28; WC - R^2^=0.27) (data not showed).

**Table 4:** Linear regression coefficients (β) and p-value for “total”, “appearance”, “weight”’ and “attribution” body esteem scores (dependent variables) regressed on body mass index and waist circumference (independent variables) for age and sex categories. Adolescents (n=305) of a public school, Rio de Janeiro, Brazil.

Independent variables	Dependent variable: body esteem							
	Appearance		Weight		Attribution^¥^		Total^¥^	
	β	p-value	β	p-value	β	p-value	β	p-value
Males								
Younger age group (10 to 14 years old; n=124)								
BMI	-0.213	0.02	-0.381	<0.01	-0.124	0.17	-0.310	<0.01
WC	-0.276	<0.01	-0.454	<0.01	-0.157	0.08	-0.383	<0.01
Older age group (15 to 18 years old; n=50)								
BMI	-0.123	0.40	-0.394	0.01	0.049	0.74	-0.244	0.09
WC	-0.110	0.45	-0.454	<0.01	0.114	0.43	-0.248	0.08
Females								
Younger age group (10 to 13 years old; n=69)								
BMI	-0.112	0.36	-0.539	<0.01	0.027	0.83	-0.298	0.01
WC	-0.178	0.14	-0.532	<0.01	-0.018	0.89	-0.335	0.01
Older age group (14 to 18 years old; n=62)								
BMI	-0.136	0.29	-0.253	0.05	0.039	0.77	-0.171	0.19
WC	-0.058	0.66	-0.223	0.08	0.058	0.65	-0.118	0.36

¥: missing data: attribution and total body esteem scores: two cases; BMI: body mass index; WC: waist circumference.

## DISCUSSION

In general, girls scored lower than boys for total and appearance and weight domains of the body esteem scale. In male adolescents younger than 15 years old, high BMI and WC were associated with low total and appearance body esteem. Furthermore, high adiposity was associated with low scores for the weight domain in both age groups among male adolescents. Among girls, high adiposity was inversely associated with total and weight body esteem only for those younger than 14 years old.

Low body esteem scores were estimated for boys younger than 15 years old classified as overweight/obese or with an elevated WC. Among older boys and younger girls considered as overweight/obese or with an elevated WC, low scores were seen only for the weight domain of body esteem. For overweight/obese girls ≥14 years old, low scores were estimated for the weight domain, as well as for appearance and total body esteem score.

Male and female Brazilian adolescents scored higher (57.2 and 53.1, respectively) on total body esteem than in a study that examined 905 Hong Kong adolescents using the same scale (boys - 42.33; girls - 40.05).^8^ ^,^ ^10^ Consistent with similar studies,^4^ ^,^ ^10^ ^,^ ^11^ in the studied group, boys tended to have higher body esteem than girls, and this relationship differed according to age group.

For overweight/obese male adolescents, low body esteem was more evident among the younger group, while these results among girls were more evident for those older than 14 years old. This finding suggests that boys and girls may respond differently to the feelings related to body image and satisfaction.^26^ Furthermore, in the first years of adolescence, gender-specific behaviors and feelings concerning body weight become more relevant.^4^ ^,^ ^27^ In linear regression models, there was no association between indicators of adiposity and body esteem scores among girls older than 14 year old, possibly due to the reduced prevalence of overweight/obesity (16.4%) and high WC (8.1%) in this group.

In the literature, we found only one study evaluating the association between central adiposity and body esteem in Brazilian adolescents. Conti, using the BESAA scale, found an inverse association between central adiposity and body esteem in the weight, appearance, and attribution domains for younger male and female adolescents.^19^

Some limitations of this study should be considered. The cross-sectional design restricts the scope for inference as to causal direction; however, longitudinal studies that have assessed weight status and global self-esteem have found that overweight/obese adolescents presented lower global self-esteem.^28^ ^,^ ^29^ ^,^ ^30^ Another limitation is the fact that the sample was made up of adolescent students from one public school in Rio de Janeiro, which limits the scope for generalizing the results to the overall population of adolescents. Furthermore, the study is based on data collected in 2009. However, literature shows that the factors determining body esteem and its repercussions on health have not changed in the last decade, for example, the increasing prevalence of overweight,^2^ ^,^ ^5^ changes in eating habits,^5^ and social pressure for the maintenance of a beauty pattern based on thinness.^6^ ^,^ ^7^ Another question may be posed by the low prevalence of underweight and high prevalence of excess weight in the studied sample, which only allowed comparisons between normal and overweight groups. Moreover, the study did not evaluate other variables that could influence the relationship between indicators of adiposity and body esteem, such as body image, socioeconomic status, and physical activity. Investigating other factors, like peer environment, may be important to understand the development of body esteem in adolescents. In addition, follow-up studies are necessary to clarify temporal changes in body esteem during the adolescence period.

This study has as strengths the use of anthropometric measurements taken with methodological care by trained and standardized researchers, which eliminates the inherent biases of the self-reported information. Additionally, the BESAA conceptualizes body esteem as a multidimensional construct, providing an understanding of the different aspects of body esteem in adolescents.^3^ ^,^ ^4^ ^,^ ^30^ The body esteem scale used in this study was designed to measure feelings about appearance, weight satisfaction, and self-perception of others’ opinions on one’s appearance and weight. This kind of evaluation is important, considering that body esteem may have a mediating role in the complex relationships between weight status, body image, and adverse mental health outcomes.^11^ According to a previous study,^3^ the BESAA has the property of being able to differentiate feelings about appearance from those about weight, allowing the identification of feelings that may affect well-being. The 23-item BESAA is easy to administer and was considered valid and reliable to be used in Brazilian adolescents: total body esteem - Cronbach’s α=0.76, appearance - Cronbach’s α=0.87, weight - Cronbach’s α=0.90, and attribution - Cronbach’s α=0.70 domains.^19^

BMI and WC, both analyzed as continuous variables as well as categorized in overweight/obesity and high central adiposity, were associated with low body esteem in adolescents, especially among the younger age groups. In both boys and girls, the BMI had an inverse relationship with body esteem. High BMI and WC were related to lower body esteem in adolescents, regardless of sex, especially in the weight domain of the body esteem scale. These findings signal the need of including the body esteem assessment in the health care of adolescents. A more comprehensive evaluation of adolescents should consider their feelings regarding their bodies and appearance. Healthy weight control must be focused on changing the conceptions about the ideal body and healthy lifestyle, rather than prioritizing losing weight through dieting.
